# Complete genome sequences of two *Treponema pallidum* subsp. *pallidum* specimens from Canadian patients

**DOI:** 10.1128/mra.00641-25

**Published:** 2025-09-18

**Authors:** Nishant Singh, Thomas W. A. Braukmann, MacKenzie Neale, George S. Long, Derek Stein, Paul Van Caeseele, Keith D. MacKenzie, Jessica Minion, Gary Van Domselaar, Morag Graham, Andrea Tyler, Samir N. Patel, Venkata R. Duvvuri, Raymond S. W. Tsang

**Affiliations:** 1 Public Health Ontario Laboratory, Public Health Ontario 153300, Toronto, Ontario, Canada; 2 Department of Laboratory Medicine and Pathobiology, University of Toronto 7938, Toronto, Ontario, Canada; 3 Syphilis Diagnostic Unit, National Microbiology Laboratory Branch, Public Health Agency of Canada, Winnipeg, Manitoba, Canada; 4 Cadham Provincial Laboratory, Winnipeg, Manitoba, Canada; 5 Roy Romanow Provincial Laboratory, Regina, Saskatchewan, Canada; 6 Bioinformatics Section, National Microbiology Laboratory Branch, Public Health Agency of Canada, Winnipeg, Manitoba, Canada; 7 Genomics & International Depository Authority of Canada (IDAC), National Microbiology Laboratory Branch, Public Health Agency of Canada, Winnipeg, Manitoba, Canada; 8 Laboratory for Industrial and Applied Mathematics, Department of Mathematics and Statistics, York University 375295, Toronto, Ontario, Canada; Wellesley College, Wellesley, Massachusetts, USA

**Keywords:** metagenomics, syphilis, *Treponema pallidum*

## Abstract

We report the genome sequences of two *Treponema pallidum* subsp. *pallidum* specimens in Canada, with basic details on the genetic diversity and antimicrobial resistance. This data contributes to the understanding of *T. pallidum* evolution and epidemiology, supporting syphilis surveillance and public health efforts.

## ANNOUNCEMENT

Syphilis, a sexually transmitted infection, is caused by the bacterium *Treponema pallidum* subspecies *pallidum* (TPA) that, when left untreated, causes a chronic, multi-stage disease (1–4). TPA is broadly divided into two monophyletic lineages, Nichols and SS14 (5). In Canada, syphilis rates have rebounded from a record low in 1999, <0.5 cases per 100,000, to 30.5 per 100,000 population in 2023 (6–9).

**Fig 1:**
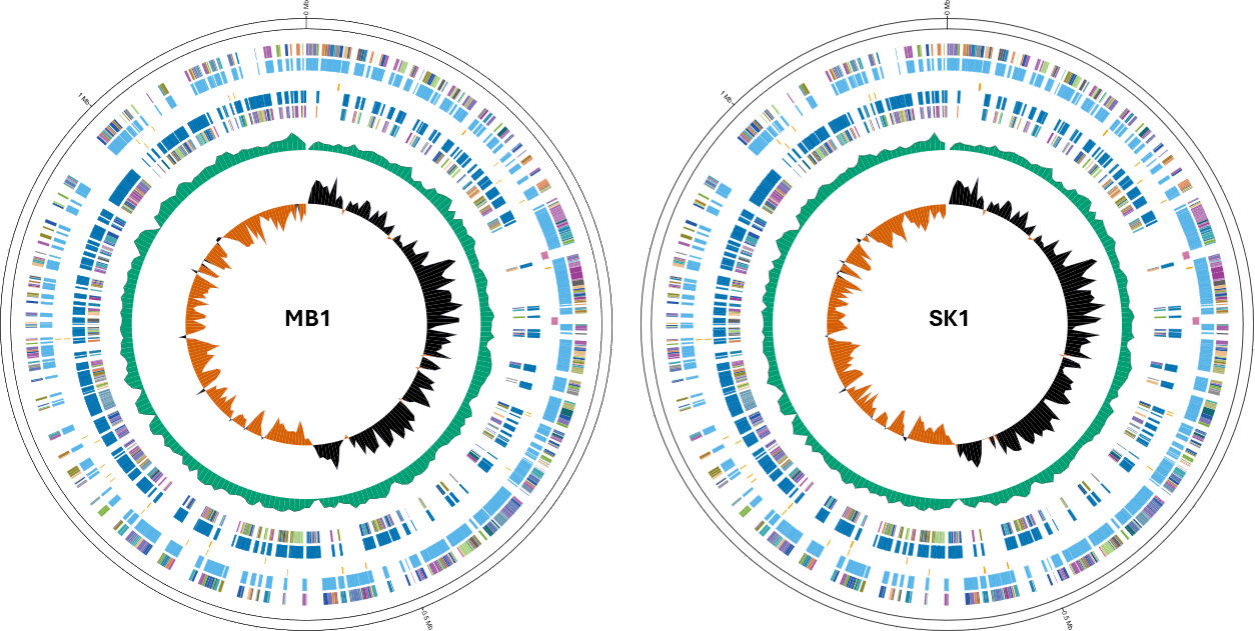
Circular genome maps for two *T. pallidum* subspecies *pallidum* samples from Canada created using GenoVi (13). From outside to inside, the map depicts the genome contig; forward strand clusters of orthologous genes (COGs); forward strandcoding sequences (CDS), tRNA, and rRNA; reverse strand CDS, tRNA, rRNA; reverse strand COGS; GC content; GC skew.

Here we report complete genome sequences of two Canadian TPA strains, MB1 and SK1, recovered in May 2016 and February 2023 from the prairie provinces of Manitoba and Saskatchewan (Fig. 1). DNA was extracted from a genital lesion for MB1 and an unknown site for SK1 using the QIAamp Kit following the manufacturer’s instructions (Qiagen, Germany). A single round of hybridization for TPA sequence enrichment was done using a custom set of SureSelect baits designed to capture both Nichols and SS-14 lineages (Agilent Technologies Canada; ELID no: 3437291). DNA was mechanically sheared into 300 bp fragments using a Covaris E220 sonicator. Fragmentation was checked on an Agilent D1000 tape station prior to bait capture (Agilent, USA; Catalog no. 5067–5582 & 5067–5583). All Illumina libraries were prepared using the Agilent SureSelect XT HS2 DNA Reagent Kit with Index Primer Pairs 1–16 (Agilent, USA) following the manufacturer’s instructions with no size selection. All libraries were checked on an Agilent D1000 tape station for DNA length distribution prior to sequencing (Agilent, USA; Catalog no. 5067–5584 & 5067–5585). For specific whole-genome amplification (SWGA), samples were amplified using the Phi29 DNA polymerase (NEB, USA) for 3 h at 45°C using a custom primer set (10) (Table 1). Both samples were sequenced on the MiSeq platform using the MiSeq v2 600-cycle reagent Kit for 300 bp paired-end reads. SWGA reads from sample SK1 were additionally sequenced on a GridION using MinION R10.4.1 flow cell with the native barcoding Kit (SQK-NBD114-24) for 72 h (Oxford Nanopore Technologies, United Kingdom). High-accuracy base calling was done using Dorado (v7.2.13) within MinKNOW (v23.11.7). Reads were filtered with chopper (v0.80), then dehosted via minimap2 (2.28-r1209) to a SS14 TPA reference (accession no. CP010565.1) (11, 12).

**TABLE 1:** Summary of genome assembly and annotation from two Canadian *T. pallidum* subspecies *pallidum* genomes from Canada^*a*^^,^^*b*^

	MB1	SK1
Completeness (%)	100	100
Contamination (%)	1.40%	1.40%
Size (bp)	1,141,273	1,139,696
GC (%)	52.76	52.77
CDS	990	985
tRNA	45	45
rRNA	3	3
Lineage	SS14	SS14
Sequence Type	1.3.1	1.3.1
AMR Predictions	resistant to azithromycin	resistant to azithromycin
GenBank accession number	CP191228	CP191227
No. Contigs >1000 bp	20	3
N50	929,486	1,139401
read depth	52	216
Illumina raw reads	3,124,109	1,247,005
Illumina Filtered reads	926,186	1,193,464
Reads filtered for SRA submission^*a*^	797,572	1,171,873
Nanopore raw reads	NA	753,535
Nanopore filtered reads	NA	377,276
% mapped to Nichols reference (mean mapping quality)	99.61	99.61
% mapped to SS14 reference (mean mapping quality)	99.94	99.95
Nanopore read N50 raw	NA	7.48 kbp
SWGA primer sequences	NA	Set 1: CGATACGTT,CGTACGAC,GTACGAAAC,TAATACGCG,TACCGGATA
	NA	Set 2: CGTAAAACG,CGTACGAC,GTACGAAAC,TAATACGCG,TATACTCGC

^*a*^ To meet privacy standards, additional filters were included to remove potential non-target sequences.

^*b*^ “NA” indicates not applicable.

Raw Illumina reads were filtered with fastp (v0.23.4), then mapped to a composite reference sequence composed of both TPA lineages (Nichols, accession no. NC_021490.2 and SS14) using BWA (v0.7.18-r1243) and samtools (v1.21) (14, 15). All mapped reads were assembled in SPAdes (v3.15.5) using a consensus sequence as a trusted contig (16). The resulting contigs from the SPAdes assembly of SK1 were mapped to the TPA SS14 reference sequence using BWA, and a consensus was generated using samtools consensus. Gaps within the draft genome of SK1 were closed using TGS-GapCloser (v1.2.1) with SWGA reads from nanopore sequencing (17). Resulting sequences were all polished with Pilon (v1.24) to correct any small single nucleotide polymorphism (SNPs) or indels (18). To check circularization of the genome, the start position was shifted to check read mapping across the start position (using BWA). The resulting genomes were annotated by NCBI with PGAP (v6.10) (19, 20). Quality of genome assemblies was determined using QUAST (v5.3.0) (21). Antimicrobial resistance (AMR) was predicted using starAMR (v0.110)(22), RGI (v6.0.3)(23), and custom queries. MLST was determined using starAMR (v0.110)(22). Genomes were visualized using GenoVi (v0.4.3)(13). Genome attributes are summarized in Table 1 and Fig. 1.

## Data Availability

This Whole Genome Sequences have been deposited in GenBank under the accession no. CP191228 and CP191227. Reads mapping to the final assemblies were deposited in the NCBI’s short read archive (SRA) under accession no. SRR34079400 for CP191228, and accession no. SRR34079399 (Illumina reads) and SRR34079399 (Nanopore reads) for CP191227.
